# Multi-Antigen Outer Membrane Vesicle Engineering to Develop Polyvalent Vaccines: The *Staphylococcus aureus* Case

**DOI:** 10.3389/fimmu.2021.752168

**Published:** 2021-11-08

**Authors:** Enrico König, Assunta Gagliardi, Ilary Riedmiller, Chiara Andretta, Michele Tomasi, Carmela Irene, Luca Frattini, Ilaria Zanella, Francesco Berti, Alberto Grandi, Elena Caproni, Laura Fantappiè, Guido Grandi

**Affiliations:** ^1^ Department of Cellular, Computational and Integrative Biology, University of Trento, Trento, Italy; ^2^ ERC Vaccibiome Unit, Toscana Life Sciences Foundation, Siena, Italy; ^3^ Technical Research and Development, GlaxoSmithKline Vaccines, Siena, Italy; ^4^ Infectious Diseases and Cancer Immunotherapy Unit, BiOMViS Srl, Siena, Italy

**Keywords:** *Staphylococcus aureus*, outer membrane vesicles (OMVs), chimeric proteins, multivalent vaccines, OMV engineering

## Abstract

Modification of surface antigens and differential expression of virulence factors are frequent strategies pathogens adopt to escape the host immune system. These escape mechanisms make pathogens a “moving target” for our immune system and represent a challenge for the development of vaccines, which require more than one antigen to be efficacious. Therefore, the availability of strategies, which simplify vaccine design, is highly desirable. Bacterial Outer Membrane Vesicles (OMVs) are a promising vaccine platform for their built-in adjuvanticity, ease of purification and flexibility to be engineered with foreign proteins. However, data on if and how OMVs can be engineered with multiple antigens is limited. In this work, we report a multi-antigen expression strategy based on the co-expression of two chimeras, each constituted by head-to-tail fusions of immunogenic proteins, in the same OMV-producing strain. We tested the strategy to develop a vaccine against *Staphylococcus aureus*, a Gram-positive human pathogen responsible for a large number of community and hospital-acquired diseases. Here we describe an OMV-based vaccine in which four *S. aureus* virulent factors, ClfA_Y338A_, LukE, SpA_KKAA_ and Hla_H35L_ have been co-expressed in the same OMVs (CLSH-OMVs_Δ60_). The vaccine elicited antigen-specific antibodies with functional activity, as judged by their capacity to promote opsonophagocytosis and to inhibit Hla-mediated hemolysis, LukED-mediated leukocyte killing, and ClfA-mediated *S. aureus* binding to fibrinogen. Mice vaccinated with CLSH-OMVs_Δ60_ were robustly protected from *S. aureus* challenge in the skin, sepsis and kidney abscess models. This study not only describes a generalized approach to develop easy-to-produce and inexpensive multi-component vaccines, but also proposes a new tetravalent vaccine candidate ready to move to development.

## Introduction

Mutation-driven modification of surface antigens and differential expression of adhesion, colonization and virulence factors are among the most frequent strategies pathogens adopt to escape the host immune system. These escape mechanisms not only make pathogens a “moving target” for our immune system but also represent a challenge for vaccine development since, to provide a sufficiently broad coverage, vaccines must be formulated with more than one, sometime several, antigens. For instance, the most recent pneumococcal and *Human Papilloma Virus* vaccines include up to 15 glycoconjugates and five variants of the L1 protein, respectively, while the acellular *Bordetella pertussis* vaccine and the *Meningococcus B* vaccine both contain five distinct virulence factors (1).

The need to formulate vaccines with more than one component complicates the production processes and increases the production costs quite substantially. Therefore, the availability of platforms, which simplify vaccine design, is highly desirable, particularly to allow broad vaccination coverage in low income countries.

Outer Membrane Vesicles (OMVs) have emerged as a promising vaccine platform which have been already exploited for human use (2). OMVs are particularly attractive for their built-in adjuvanticity, which avoids the need of additional adjuvants to elicit antigen-specific immune responses (3). Moreover, OMVs can be easily purified: OMV purification essentially consists in the separation of the biomass from the culture supernatant and in the use of tangential flow ultrafiltration to purify and concentrate the released vesicles from the latter (4). Finally, OMVs can be decorated with foreign proteins/polypeptides by genetic manipulation of the OMV-producing strains (5–7) and it has been extensively shown that immunization with engineered OMVs induce potent antigen-specific immune responses (8, 9).

OMV engineering has been proven for single antigens and therefore the preparation of multi-component vaccines requires the purification and the subsequent combination of individual OMVs (9). Although OMVs are very easy to produce, the co-expression of more than one antigen in the same OMVs would simplify the production of multivalent vaccines additionally. However, so far data on if and how OMVs can be engineered with multiple full-length antigens are limited. We previously published the decoration of OMVs with a string of immunogenic epitopes (10) and Daleke-Schermerhorn et al. (11) used the Hbp autotransporter to deliver to the OMV surface protein fusions constituted by up to three small size antigens/protein domains.

In this work, we have investigated the possibility to decorate OMVs with more than one antigen and we describe a strategy to co-express four antigens in the same OMVs. We also show that the immunization of mice with four-antigen OMVs elicit functional immune responses against each engineered antigen.

To test the feasibility and the effectiveness of our multivalent OMV approach, we focused our attention on *Staphylococcus aureus*. This pathogen appears to be an attractive model since it expresses more than 35 immune evasion molecules and various virulence factors (12) and it is generally accepted that to be effective a vaccine should contain a selected combination of antigens (9, 13, 14). Moreover, once phagocytosed, *S. aureus* has the ability to avoid killing, thus using phagocytes as “Trojan Horses” to disseminate itself inside the host (15, 16). Therefore, to counteract the ability of *S. aureus* to survive inside host cells, a vaccine should elicit a Th1/Th17-skewed adaptive immune response and strong innate immunity, a property that is intrinsic to OMVs.

Here we describe the co-expression, in the proteome-minimized OMVs released by *E. coli* BL21(DE3)Δ60 (17), of ClfA_Y338A_, LukE, SpA_KKAA_ and Hla_H35L,_ four well characterized virulent factors shown to induce protection in different animal models. The vaccine (CLSH-OMVs_Δ60_) elicits antigen-specific antibodies with functional activity, as judged by their capacity to promote opsonophagocytosis and to inhibit Hla-mediated hemolysis, LukED-mediated leukocyte killing, and ClfA-mediated *S. aureus* binding to fibrinogen. Mice vaccinated with CLSH-OMVs_Δ60_ are robustly protected from *S. aureus* challenge in the skin, sepsis and kidney abscess models.

This study provides a generalized approach to develop easy-to-produce and inexpensive multi-component vaccines. Moreover, considering that the four selected virulence factors are among the most promising protective antigens for *S. aureus*, this study proposes a new tetravalent vaccine candidate ready to move to development.

## Materials and Methods

### Bioinformatics Analysis and Software

Oligonucleotides (primers) were designed using Serial Cloner and Benchling. Densitometry was performed with Image Studio Lite Version 5.2 (Licor).

### Bacterial Strains, Culture Conditions and Plasmids

*E. coli* strains (BL21(DE3) derivatives, DH5α, TOP10, HK100) were routinely cultured in lysogeny broth (LB Miller; Sigma-Aldrich) supplemented with ampicillin (100 mg/L), chloramphenicol (25 mg/L) or a combination of both, when necessary. For recombinant protein expression in OMVs, cultures were supplemented with 0.1 mM isopropyl-β-D-thiogalactopyranoside (IPTG) at OD_600_ between 0.5 and 0.7 and OMVs were collected after 3 hours from induction. Fermentation was carried out using an ez-Control bioreactor (Applikon) at 30°C until OD_600_ 0.5, then growth continued at 25°C, pH 6.8 ( ± 0.2), dO_2_ > 30%, 280-500 rpm. At OD_600_ 1.0, the culture was induced with 0.1 mM IPTG together with a feed consisting in ampicillin (50 mg/L) and/or chloramphenicol (12.5 mg/L), glycerol (15 g/L), and MgSO_4_ (0.25 g/L).

Plasmid derivatives of the pET21b(+) backbone encoding FhuD2, SpA_KKAA_, LukE, Hla_H35L_, ClfA_Y338A_ were already described (9, 17), while derivatives of the pACYC backbone were constructed *ex novo* using PIPE-PCR (18). Briefly, the coding sequence of the target protein was PCR amplified from its respective template pET21b(+) plasmid (I-PIPE) using the primers reported in **Supplementary Table 1**. These primers feature overhangs that are complementary to the 5’- and 3’-ends of a linearized “recipient” pACYC derivative. The latter plasmid was linearized by PCR (V-PIPE) using the primer couples listed in **Supplementary Table 1**. The PCR products of both reactions were digested with DpnI (New England BioLabs), combined (2 μl each) and used to transform chemically competent *E. coli* HK100 cells. Positive clones identified by colony PCR using GoTaq^®^ Green Master Mix (Promega) and gene specific primers (**Supplementary Table 1**) were subjected to sequence analysis (Eurofins) before using the resultant plasmids (**Supplementary Table 2**) for transformation of the OMV producer strain.

The PIPE-PCR method (18) was also used to construct plasmids encoding chimera. Here, the coding sequence of the C-terminal protein of the chimera was PCR amplified from its respective template plasmid (I-PIPE) using the primers reported in **Supplementary Table 1**. These primers feature overhangs that, at the 5’-end, encode a short linker sequence (translated as GGGGS) complementary to the 3’-end of a “recipient” pET21b(+) derivative carrying the Lpp leader sequence followed by the N-terminal antigen of the chimera, while at the 3’-end the overhangs carry the stop codon and are complementary to the 5’-end of the recipient plasmid. The recipient plasmids were linearized by PCR (V-PIPE) excluding the stop codon and introducing the same linker sequence stated above using the primer couples listed in **Supplementary Table 1**. The PCR products were mixed together as described above and the resultant plasmids (**Supplementary Table 2**) were used for transforming the OMV producer strain.

### Expression and Purification of Recombinant Proteins

FhuD2, LukE, SpA_KKAA_ and Hla_H35L_ *S. aureus* proteins were purified as previously described (9).

For ClfA_Y338A_, the gene coding for the wild-type N1N2N3 portion (amino acid 40 to 559) of the antigen was amplified from the genomic DNA of *S. aureus* Newman strain. After genomic amplification, the Y338A mutation was inserted using PIPE-PCR. In particular, the gene was first amplified using primer couples 32 + 37 and 36 + 33 (**Supplementary Table 1**) and then the PCR product was used as template for the final PCR with the primer 32 + 33. The resulting gene (*clfA_Y338A_*) was then cloned in a pET15 plasmid (containing His_6_-tag and TEV cleavage site coding sequences) downstream from a T7 inducible promoter and expressed in *E. coli* BL21(DE3) strain. Bacterial biomass was produced using an ez-Control bioreactor (Applikon Biotechnology) and purification of the recombinant protein was performed using an ÄKTA purifier System (General Electric Healthcare) for IMAC and size-exclusion chromatography and 10 mM Tris + 100 mM NaCl and PBS (both pH 8.0), respectively, as buffer.

### OMV Purification and Analysis

OMVs were purified as previously described (9, 17, 19). Purification from cultures in bioreactor was carried out using Tangential Flow Filtration (ÄKTA flux system, GE Healthcare) with a Hollow Fiber cartridge UFB-500-C-3MA (GE Healthcare). Protein content of OMVs was quantified using DC™ Protein Assay (Bio-Rad) and the quality of OMVs was monitored by SDS-PAGE loading OMVs (normalized by μg) on Any kD™ Criterion™ TGX Stain-Free™ Protein Gel (BioRad) stained with ProBlue Safe Stain (Giotto Biotech). The size of purified OMVs was determined using NanoSight NS300 (Malvern Panalytical Ltd) as previously described (17).

### Triton X-114 Protein Separation From OMVs and Western Blot Analysis

OMVs (100 μg of proteins) were diluted in PBS, ice cold Triton X-114 was added to 1% final concentration and the OMV-containing solution was incubated at 4°C for 1 hour under shaking. The solution was then heated at 37°C for 10 minutes and the aqueous phase was separated from the detergent phase by centrifugation at 13,000 g for 10 minutes. Proteins in both phases were then precipitated by standard chloroform/methanol procedure, separated by SDS-PAGE electrophoresis and the protein of interest visualized by Western blot. Western blot analysis was performed as previously reported (9). Antibodies against each antigen were obtained from Genscript by immunizing rabbits with the following antigen-specific synthetic peptides: LukE: NEFVTPDGKKSAHD, SpA_KKAA_: AKKLNDAQAPKADN, Hla_H35L_: GTNTKDKWIDRSSE, ClfA_Y338A_: IDKPVVPEQPDEPG. All synthetic peptides were conjugated to KLH protein.

### Mice Immunization, Challenge and ELISA

Animal experiments were carried out in accordance with experimental protocols that were reviewed and approved by the Animal Ethical Committees of the University of Trento and Toscana Life Sciences (Siena, Italy) and by the Italian Ministry of Health.

For antibody titers, five-week old CD1 female mice were immunized intraperitoneally (i.p., 100 μl final volume) and intramuscularly (i.m., 50 μl final volume) three times every two weeks with 20 μg of CLSH-OMVs_Δ60_ formulated either with or without Alum hydroxide (2 mg/ml). Blood was collected from anesthetized mice through cardiac puncture at day 35 and serum was obtained from blood through centrifugation at 2,000 rpm for 10 minutes.

For challenge studies, mice were immunized i.p. at day 0, 14 and 28 with either 20 μg of “empty” OMVs_Δ60_ or 20 μg of CLSH-OMVs_Δ60_ in the presence of 2 mg/ml Alum. Alum was included in the vaccine formulation to use conditions similar to Bexsero, a vaccine which contains OMVs and has been approved for human use (2). For the sepsis model of infection two weeks after the third immunization mice were i.p. challenged with 3×10^8^ CFUs of *S. aureus* Newman strain. Mice were monitored daily for a 7-day period. Animals health was evaluated using a 1 to 4 pain scale. A value of 4 was given to mice with: loss of weight >15%, very rough hair coat, impaired mobility. A score of 3 was given to mice with loss of weight approximately 15% and rough hair coat, while scores of 2 and 1 were given to mice with a loss of weight between 6% and 14% or 0% and 5%, respectively. All procedures were approved by the National Health Institution and the Ethical Committee and for human reasons animals were sacrificed at symptoms of sickness as recommended by 3Rs rules (‘‘Refinement, Reduction, Replacement’’ policy towards the use of animals for scientific procedures_ 99/167/EC, Council Decision of 25/1/99).

Experiments using the renal abscess and skin infection model were performed as previously described (13). Briefly, for the renal model mice were challenged intravenously (i.v.) 10 days after the third immunization with 1×10^7^ CFUs of *S. aureus* Newman strain, while in the skin infection model mice were challenged subcutaneously (s.c.) 14 days after the third immunization with 5×10^7^ CFUs of *S. aureus* Newman strain.

ELISA assays on mice sera collected after immunizations were performed as previously described (9) using alkaline phosphatase-conjugated secondary antibodies that recognize murine total IgG (Sigma-Aldrich), IgG2a or IgG1 (both Biolegend).

### Functional Assays

#### Opsonophagocytosis Killing (OPK) Assay

The OPK assay was performed following previously published protocols (20–22). In detail, *S. aureus* Newman strain was grown in tryptic soy broth (TSB) under aerobic and shaking conditions (170 r.p.m.) at 37°C starting from OD_600_ 0.05 for 1 hour. Then, bacteria were harvested by centrifugation and diluted in Reaction Buffer (RB, DMEM + BSA 1%) and 500 CFUs/15µl were incubated with previously serially diluted immune sera for 45 minutes at room temperature (RT) under shaking (550 r.p.m.). Human promyelocytic leukemia HL-60 cells (ATCC CCL-240), differentiated into neutrophils by adding 0.78% (v/v) dimethylformamide (DMF) and incubating cells at 37°C and 5% CO_2_ for 5 days, were adjusted to 3×10^5^ cells in 15µl RB, and Low-Tox Guinea Pig complement (Cederlane) diluted in RB to 4% (15µl) was added. As negative controls we included the following: i) Bacteria + RB without (w/o) serum + HL-60 cells + complement, ii) Bacteria + RB w/o serum and w/o HL-60 cells + complement, iii) Bacteria + RB w/o serum + HL-60 cells + heat-inactivated complement (HC), and iv) the same as in iii) + tested serum at the lowest dilution (1:100). The reaction mixtures were incubated at 37°C with shaking (550 r.p.m.). After 90 minutes (T90), the reactions were stopped by incubation on ice for 5 minutes and addition of 15 µl 1% Triton X-100 to facilitate cell lysis and bacterial release to the supernatant. After 3 minutes of incubation at 37°C with agitation, 10 µl of each mixture were diluted in RB (1:5) and plated on tryptic soy agar (TSA) plates. Bacterial CFUs were counted manually after an overnight incubation at 37°C. Bacterial OPK for each sample was calculated with respect to the CFUs of the negative control containing bacteria + HL-60 + complement without serum according the following formula: *[CFUs of the negative control at T90 - CFUs of each sample at T90]/CFUs of the negative control at T90*.

#### ClfA Neutralization Assay

Flat-bottom 96-well plates (Nunc MaxiSorp™) were coated with 10 μg/ml human fibrinogen (Merck) overnight at 4°C. The plates were blocked with BSA 5% (p/v) for 2 hours at 37°C and then washed 3 times with PBS. *S. aureus* Newman strain (1×10^7^ CFUs), which was grown in TSB to mid-log phase under aerobic and shaking conditions at 37°C, was pre-incubated with serially diluted immune sera for 30 minutes at RT and then transferred to the fibrinogen-coated plates. After 1-hour incubation at 37°C, adherent cells were washed once with PBS, fixed with formaldehyde 2.5% (v/v) for 30 minutes at RT and stained with crystal violet (CV) 0.5% (v/v) for 10 minutes at RT. Bacteria were then washed once with PBS and the plates were allowed to air-dry. CV was solubilized using acetic acid 30% (v/v) for 15 minutes at RT. Then, the crystal violet/acetic acid solution was transferred from each well to a separate well in a flat-bottom 96-well plate. Absorbance was read at 595 nm using a SpectraMax M2 Microplate reader (Molecular Devices). Adherence of tested samples was given as a percentage of values measured in wells lacking serum (100% adherence), and the percentage of inhibition of binding to fibrinogen was calculated by subtracting the adherence percentage values from 100.

#### LukED Neutralization Assay

LukE neutralization was assayed using the Cell proliferation kit II (XTT, Sigma-Aldrich). HL-60 cells were cultured in flask in RPMI medium plus 10% fetal bovine serum (FBS), 100 U/ml penicillin and 100 μg/ml streptomycin, 2mM glutamine, 1mM Sodium pyruvate and non-essential amino acids at 37°C and 5% CO_2_. Then, HL-60 cells were differentiated into neutrophils by adding 0.78% (v/v) dimethylformamide (DMF) and incubating cells at 37°C and 5% CO_2_ for 5 days. Subsequently, 5×10^5^ differentiated HL-60 cells in a volume of 10 μl/well of RPMI culture medium were dispensed into flat-bottom 96-well plates (Corning, Corning, NY USA) and incubated with either rLukE, or rLukD (Abcam) as negative control or LukED (200 nM in 40 μl/well of RPMI culture medium) in presence of diluted immune sera (50 μl/well), for 24 hours at 37°C. Cell viability was then measured after 16 hours incubation with XTT Assay reagent (50 μl/well; Sigma-Aldrich) according to manufacturer’s instructions by reading the absorbance at 470nm using a SpectraMax M2 Microplate reader (Molecular Devices).

#### Hla Neutralization Assay

Hla neutralization assay was performed as previously described (9).

## Results

### Rationale of *S. aureus* Antigen Selection

To test the possibility of designing a multivalent vaccine constituted by a single preparation of *E. coli*–derived OMVs engineered with four heterologous proteins, we focused our attention on SpA_KKAA_, ClfA_Y338A_, Hla_H35L_ and LukE antigens.

SpA substantially contributes to *S. aureus* virulence and toxicity. By binding to the Fcγ domain of antibodies, surface-exposed SpA interferes with the association of the C1q component of the complement classical pathway, and this results in the inhibition of bacterial opsonophagocytosis and killing (OPK) by phagocytic cells (23). Moreover, by cross-linking V_H_3 clonal B cell receptors, secreted SpA acts as a B cell super-antigen promoting the production of all V_H_3 antibodies, irrespectively of their antigen specificity (24). Finally, SpA is responsible for an anaphylactic syndrome due to its binding to the V_H_3 region of IgG and IgE antibodies associated to basophils and mast cells (25). It was elegantly shown that immunization with the SpA_KKAA_, a mutant no longer capable of binding Fcγ and V_H_3, induces antibodies which (i) inhibit SpA binding to Ig, (ii) promote OPK in mouse, guinea pig and human blood (23, 26), and (iii) protect animals from bacteremia (27).

Clumping Factor A (ClfA) is a surface-exposed virulence factor expressed in most *S. aureus* isolates and whose primary function is to allow the adhesion of *S. aureus* to fibrinogen (28–32). Since fibrinogen is ubiquitous in the host, ClfA promotes a rapid attachment of bacteria to tissues and organs, thus playing a relevant role in the initiation of infection (33). Inhibiting the fibrinogen binding capacity of ClfA has been proposed as a way to prevent pathogenesis. Indeed, immunization of monkeys and human volunteers with the N1N2N3 portion of ClfA carrying the Y338A mutation (30) elicited high titers of antibodies which prevented *S. aureus* adhesion to fibrinogen (32).

*S. aureus* α-hemolysin (Hla) (also known as α-toxin) is a pore-forming β-barrel toxin (34, 35), which assembles in a heptameric structure forming a pore of 2-nm in diameter into the plasma membrane (35). The toxin is expressed by almost all clinical isolates (36) and its level of expression correlates with increased severity of Skin and Soft Tissue Infections (SSTIs) and pneumonia in humans (37, 38). Hla causes dermonecrotic skin injury by interacting with ADAM10, a zinc-dependent metalloprotease that cleaves E-cadherin and destabilizes the epithelial barrier upon toxin binding (39, 40). The key role of Hla in *S. aureus* pathogenicity is highlighted by experiments showing that the intraperitoneal injection of 10 μg of purified Hla is sufficient to kill mice (41) and the passive transfer of neutralizing monoclonal antibodies is highly protective against *S. aureus* infection in different animal models (42, 43). Finally, vaccination with the nontoxigenic Hla_H35L_ mutant (44) protects mice against *S. aureus*-mediated pneumonia (37), lethal sepsis, organ dissemination (45) and skin infection (13).

Human *S. aureus* isolates produce five additional pore-forming toxins known as leukocidins (46). Leukocidins are constituted by two subunits, the host cell targeting S component and the polymerization F component. The major target of leukotoxins are immune cells of myeloid and lymphoid lineages, suggesting that these toxins have evolved to inhibit both innate and adaptive immunity. Among the five leukocidins, LukED appears to be particularly important in that it kills virtually all immune cells, including neutrophils, monocytes, macrophages, dendritic cells, T cells, erythrocytes and NK cells (46). The toxin is encoded in the stable *S. aureus* pathogenicity island vSaβ53, which is present in about 70% of all clinical isolates (47). Because of their key role in pathogenesis, inactivated leukocidins are considered to be key targets for both therapeutic and prophylactic intervention (46, 48).

Based on the above, a vaccine including SpA_KKAA_, Hla_H35L_, ClfA_Y338A_ and LukE should elicit immune responses that, in the presence of innate immune responses promoted by the OMVs, should synergize and confer protection against *S. aureus* infections. Therefore, we selected these four antigens to attempt the construction of a multivalent vaccine based on a single OMV preparation.

### OMVs Can Be Engineered With Two Antigen-Chimeras

**Figure 1:**
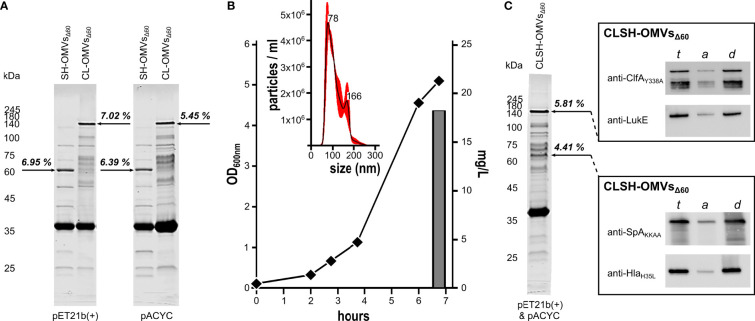
Expression of Staphylococcal antigen fusions in OMVs from *E. coli* BL21(DE3)Δ60. **(A)** OMVs from *E. coli* BL21(DE3)Δ60 strains expressing heterologous antigen fusions SpA_KKAA_-Hla_H35L_ (SH-OMVs_Δ60_) and ClfA_Y338A_-LukE (CL-OMVs_Δ60_) as lipoproteins using either pET21b(+) or pACYC as plasmid backbone were purified from culture supernatants as described in Materials and Methods. Aliquots (10 μg of total OMV proteins) were analyzed by SDS-PAGE. **(B)** Growth curve, OMV production yield and particle size analysis of CLSH-OMVs_Δ60_ grown in a 3-L bioreactor (see *Materials and Methods* for details). The size of CLSH-OMVs_Δ60_ was determined using a NanoSight NS300 (Malvern Panalytical). The graph shows the average of three independent measurements and standard error as calculated by the manufacturer’s software. Numbers indicate the particle size of main peaks. **(C)** OMVs from *E. coli* BL21(DE3)Δ60 co-expressing the two heterologous antigen fusions ClfA_Y338A_-LukE and SpA_KKAA_-Hla_H35L_ as lipoproteins (CLSH-OMVs_Δ60_) and analysis of protein lipidation by Triton X-114 extraction of OMV proteins. ClfA_Y338A_-LukE and SpA_KKAA_-Hla_H35L_ were co-expressed using pET21b(+) and pACYC, respectively, and purified and analyzed by SDS-PAGE as stated for panel **(A)** For analysis of protein lipidation, CLSH-OMVs_Δ60_ expressing heterologous protein fusions in the membrane were dissolved by adding 1% Triton X-114 at 4°C and subsequently aqueous and detergent phases were partitioned by centrifugation. Unfractionated total proteins from CLSH-OMVs_Δ60_ (*t*), hydrophilic proteins in the aqueous phase (a), and hydrophobic proteins in the detergent phase (d) were precipitated with chloroform-methanol and separated by SDS-PAGE. Finally, proteins were transferred onto nitrocellulose filters and the presence of the heterologous antigen fusions in either the aqueous or detergent phase was detected by Western blot using antigen-specific antibodies. The numbers next to each arrow **(A, C)** represent the percentage of each recombinant chimera over total OMV protein content as estimated by densitometry using Image Studio Lite Ver 5.2.

Supported by preliminary data indicating that even high molecular weight heterologous antigens can be efficiently incorporated in OMVs, we created two chimeras carrying the lipoprotein leader sequence of Lpp at their 5’-ends (9) by fusing ClfA_Y338A_-LukE genes and SpA_KKAA_-Hla_H35L_ genes. The gene fusions were cloned in both pET21b(+) and pACYC, two compatible plasmids, which differ in copy number. The plasmids were used to transform the hyper-vesiculating strain *E. coli* BL21(DE3)Δ60 recently created in our laboratories (17). OMVs were purified from the culture supernatants and analyzed by SDS-PAGE. As shown in **Figure 1A**, both chimeras accumulated in the vesicular compartment at a level of 7% of total OMV proteins, as determined by densitometry scanning. Interestingly, the different copy numbers of pET21b(+) and pACYC did not substantially influence the level of accumulation in the vesicular compartment, suggesting that the rate limiting step of chimera compartmentalization in OMVs was largely determined by the translation and/or transport machineries.

### OMVs Can Be Engineered With Four Antigens by Co-Expressing Two Chimeras

pET21b(+) and pACYC plasmids have compatible origins of replication. This prompted us to test whether the co-transformation of *E. coli* BL21(DE3)Δ60 with two recombinant plasmids, one having the pACYC scaffold and the other the pET21b(+) scaffold, could lead to the co-expression of the two chimeras in the OMVs. *E. coli* BL21(DE3)Δ60 was co-transformed with the plasmid couple pET(ClfA_Y338A_-LukE)/pACYC(SpA_KKAA_-Hla_H35L_). The recombinant strain was grown in liquid culture using a bioreactor, and after 4-hour induction of protein expression with 0.1 mM IPTG, the OMVs, named CLSH-OMVs_Δ60_, were purified from the culture supernatant (**Figure 1B**). Particles sizes and protein content of CLSH-OMVs_Δ60_ were analyzed by NanoSight NS300 and SDS-PAGE, respectively. CLSH-OMVs_Δ60_ had a mean size of 111.6 +/- 2.1 nm, in line with the size of “empty” OMVs_Δ60_ previously reported (17) (**Figure 1B**). As shown in **Figure 1C**, two protein bands corresponding to the molecular mass of the two chimeras were visible among total OMV proteins. Based on densitometry analysis, the two co-expressed chimeras accumulated in similar amounts, corresponding to 4.4% and 5.8% of total proteins from SpA_KKAA_-Hla_H35L_-OMVs_Δ60_ and ClfA_Y338A_-LukE-OMVs_Δ60_, respectively.

The fusion of the two chimeras to the lipoprotein leader sequence of Lpp should promote their N-terminal lipidation and subsequent compartmentalization in the OMV membrane (9). To indirectly demonstrate the presence of the acyl groups at the N-terminus of the two chimeras, CLSH-OMVs_Δ60_ were solubilized at 4°C with a 1% water solution of Triton X-114 and subsequently the sample was warmed to 37°C to partition Triton X-114 into two phases: a detergent-rich ‘hydrophobic’ phase and a detergent-poor ‘hydrophilic’ phase. Membrane proteins, including lipoproteins, typically partition into the hydrophobic phase (49). Aliquots from both aqueous and organic phases were analyzed by Western Blot using antibodies specific for the corresponding *S. aureus* antigens. As shown in **Figure 1C**, the two chimeras compartmentalized in the hydrophobic phase.

### CLSH-OMV_Δ60_ Immunization Elicited Functional Antigen-Specific Antibody Responses

**Figure 2:**
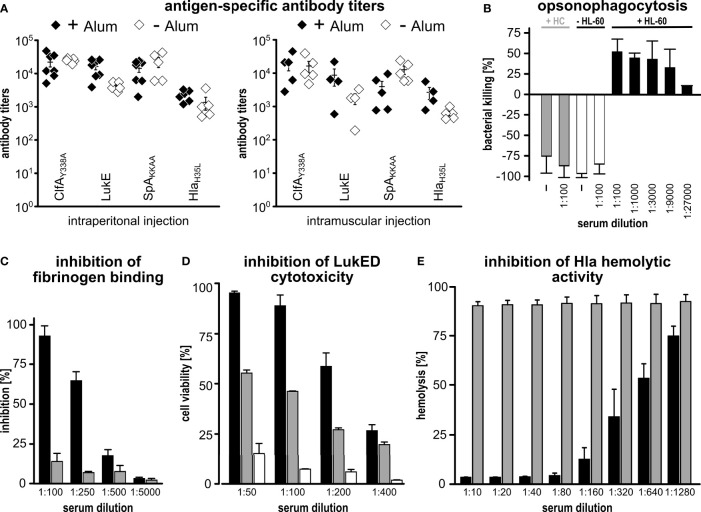
Immunogenicity of CLSH-OMVs_Δ60_. **(A)** *Antigen-specific antibody titers.* Groups of 8 female CD1 mice were immunized i.p. (left panel) or i.m. (right panel) 3 times at 2-week intervals with 20 μg of CLSH-OMVs_Δ60_ formulated with or without Alum (filled and open symbols, respectively). Sera were collected 7 days after the last immunization and IgG titers were analyzed by ELISA using plates coated with the respective purified recombinant protein (300 ng/well). Each data point represents the antibody titer from a single mouse. Mean ± s.e.m. is shown. **(B)** *Opsonophagocytosis killing (OPK).* 500 CFUs of *S. aureus* Newman strain grown in TSB at 37°C were incubated with serially diluted immune sera for 45 minutes at RT. Then, 3×10^5^ HL-60 cells differentiated into neutrophils and guinea pig complement were added to each reaction for 90 minutes at 37°C (T90). Bacterial killing refers to the CFUs of the negative control (bacteria + HL-60 cells + complement without serum) set as 0% killing and was calculated as follows: *[CFUs of the negative control at T90 - CFUs of each sample at T90]/CFUs of the negative control at T90]*. Data are reported as mean ± s. d. of two independent experiments. HC: heat-inactivated complement. **(C)** *Inhibition of fibrinogen binding*. *S. aureus* Newman strain (1×10^7^ CFUs/well) was pre-incubated for 30 minutes at RT with serially diluted pooled sera from mice immunized with either 20 μg of “empty” OMVs_Δ60_ (grey bars) or 20 μg of CLSH-OMVs_Δ60_ (black bars). Mixtures were transferred to a 96-well plate previously coated with human fibrinogen (10 µg/ml) and incubated for 1 hour at 37°C. Adherent bacteria were fixed with formaldehyde and then stained with crystal violet. Adherence to fibrinogen was calculated as a percentage of values measured in control wells lacking serum (= 100%), and inhibition of binding to fibrinogen was calculated by subtracting the adherence percentage values from the control (= 100% - x %). The graph shows the mean ± s. d. of three independent experiments. **(D)** *Inhibition of leukocidin-mediated cytotoxicity*. HL-60 cells differentiated into neutrophils (5×10^5^/well) were incubated for 24 hours at 37°C with LukED (200 nM) in presence of serially two-fold diluted sera from mice immunized with 20 μg of CLSH-OMVs_Δ60_ (black bars), “empty” OMVs_Δ60_ (grey bars) or with Alum (white bars). For assessment of cell viability, the XTT Assay reagent was added and after 16-hour incubation at 37°C the absorbance was read at 470 nm. Data are reported as mean ± s. d. of two independent experiments. **(E)** *Inhibition of Hla-mediated hemolysis*. Serially two-fold diluted sera from mice immunized with either 20 μg of “empty” OMVs_Δ60_ (grey bars) or 20 μg of CLSH-OMVs_Δ60_ (black bars) were pre-incubated with recombinant Hla for 20 minutes at RT and then with rabbit erythrocytes for 30 minutes at 37°C. Hla hemolytic activity was calculated as percentage of hemolytic activity obtained for rabbit erythrocytes incubated with water (100% hemolysis). The graph shows the mean ± s. d. of three independent experiments.

Next, we tested whether the immunization with CLSH-OMVs_Δ60_ could elicit antigen-specific antibody responses. Mice were immunized intraperitoneally (i.p.) three times, two weeks apart, with 20 μg of CLSH-OMVs_Δ60_ + Alum and we determined the antibody titers against each antigen by ELISA using plates coated with the respective purified recombinant proteins. As shown in **Figure 2A**, immunization elicited antibodies specific for each of the four *S. aureus* antigens present in the CLSH-OMVs_Δ60_, while in sera from mice immunized with “empty” OMVs no antigen-specific antibodies were detected (**Supplementary Figure 1**).

We also tested the role of Alum and the route of immunization in determining the level of antibody titers. To this aim, we repeated the i.p. immunization in the absence of Alum, and we immunized mice intramuscularly (i.m.) with 20 μg of CLSH-OMVs_Δ60_ in the presence or absence of Alum. Sera from immunized mice were analyzed by ELISA (**Figure 2A**). In the presence of Alum, the route of immunization did not substantially influence the antibody titers, with the exception for SpA_KKAA_ antibody titers, which were approximately 3.5-fold lower when the vaccine was administered intramuscularly. As far as the influence of Alum is concerned, only a marginal difference was observed when mice were immunized i.p., while titers against Hla_H35L_ and LukE were enhanced by Alum in i.m.-immunized mice. Finally, IgG titers against SpA_KKAA_ appeared to be higher in the absence of Alum when the i.m. route was used.

Next we evaluated the IgG isotypes as an indication of the type of immune response (Th1 vs. Th2-skewed response) induced by CLSH-OMVs_Δ60_ immunization. The sera from animals immunized i.p. in the presence of Alum were analyzed by ELISA using anti-mouse anti-IgG2a and anti-IgG1 antibodies as secondary antibodies and coating plates with ClfA_Y338A_ and SpA_KKAA_, the two N-terminal antigens of the chimeras. As shown in **Supplementary Figure 2**, and in line with what previously observed (8), IgG2a were abundantly present in the sera of mice immunized with CLSH-OMVs_Δ60_, indicating that the immunization elicited a Th1-skewed response.

Finally, we tested whether the antigen-specific antibodies induced by immunization with CLSH-OMVs_Δ60_ had functional activities. First, we analyzed the capacity of differentiated HL-60 cells to kill the *S. aureus* Newman strain in the presence of different dilutions of mouse sera and guinea pig complement. As shown in **Figure 2B**, the sera from mice immunized with CLSH-OMVs_Δ60_ rescued the HL-60-mediated bacterial killing in a dose-dependent manner.

Next, we tested whether the same sera could inhibit the biological activity of ClfA, LukED and Hla. To follow ClfA inhibition, plates were coated with fibrinogen and the binding of *S. aureus* Newman strain was analyzed in the presence or absence of sera from CLSH-OMVs_Δ60_-immunized mice. In the case of LukED, a toxin which can kill activated HL-60 cells, anti-CLSH-OMVs_Δ60_ sera and HL-60 cells were incubated together with purified LukED. Finally, the inhibition of the hemolytic activity was tested by incubating the sera with purified Hla and rabbit erythrocytes. As shown in **Figures 2C–E**, the pool of mouse sera from the CLSH-OMVs_Δ60_-immunized group inhibited the activities of all three virulence factors. In the case of LukED inhibition, “empty” OMVs_Δ60_ gave some neutralization, but well below the inhibition of sera from CLSH-OMV_Δ60_-immunized mice. This effect could be due to the presence of some cross-reacting antibodies elicited by OMVs_Δ60_.

### CLSH-OMVs_Δ60_ Effectively Protected Mice From the Challenge With Newman Strain

**Figure 3:**
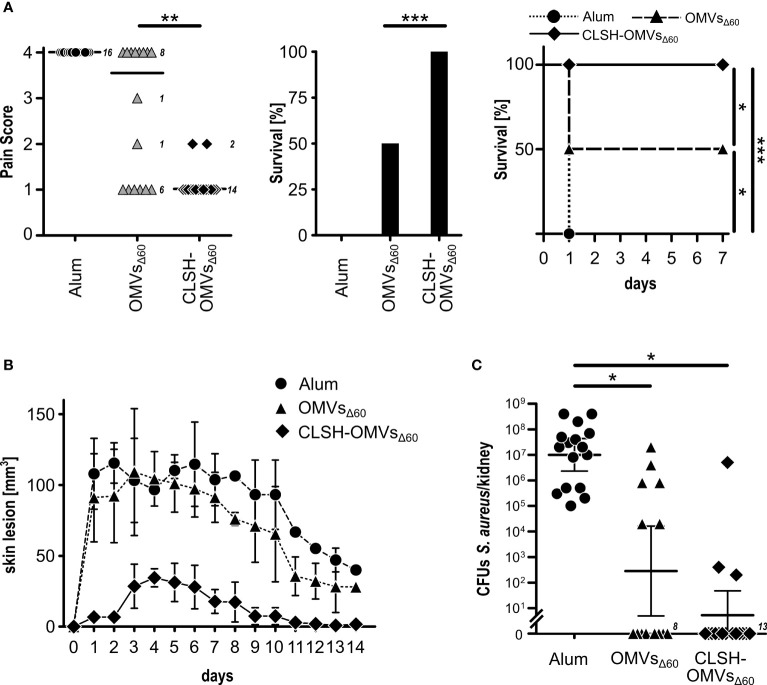
*In vivo* protective activity of OMVs_Δ60_ expressing *S. aureus* antigens. **(A)** *Sepsis model*. Groups of 16 female CD1 mice were immunized three times at 2-week intervals with Alum alone (circles), “empty” OMVs_Δ60_ (triangles) or CLSH-OMVs_Δ60_ (diamonds), formulated in Alum. After 2 weeks, mice were infected i.p. with 3×10^8^ CFUs of *S. aureus* Newman strain. Animal health was monitored over a period of 7 days, assigning a “pain score” from 1 to 4. Animals which reached a pain score = 4 were sacrificed. The three graphs show the pain score (left), survival at day 7 (center) and the survival over time (Kaplan-Meier curve) (right). See text for definition of “survival”. Statistical analysis for the pain score plot was performed using Mann-Whitney test. Median is shown. **P = 0.0015. Statistical analysis for the Kaplan–Meier plot was performed using the log rank test. *P = 0.0253; ***P = 0.0001. **(B)** *Skin model*. Groups of 16 CD1 female mice were immunized three times at 2-week intervals with Alum alone (circles), “empty” OMVs_Δ60_ (triangles) and CLSH-OMVs_Δ60_ (diamonds). At day 14 after the third immunization, mice were s.c. infected with 5×10^7^ CFUs of *S. aureus* Newman strain. Abscess size was monitored once a day for 14 days. Mean ± s. d. is shown. Skin abscess areas of vaccinated and control animals were tested for significance by means of repeated ANOVA measures using the R base function *aov()*. **(C)** *Renal abscess model*. Groups of 16 CD1 female mice were immunized three times at 2-week intervals with Alum alone (circles), “empty” OMVs_Δ60_ (triangles) and CLSH-OMVs_Δ60_ (diamonds). Ten days after the last immunization, mice were infected i.v. with a sub-lethal dose of *S. aureus* Newman strain (1×10^7^ CFUs) and 4 days afterward, mice were sacrificed, kidneys collected and homogenized in PBS, and aliquots were plated on agar media for CFUs determination. Statistical analysis was performed using two-tailed Student’s t-test. Geometric mean ± 95% confidence interval is shown. *P < 0.05.

Finally, we followed the protective activity of CLSH-OMVs_Δ60_ immunization in mice challenged with *S. aureus* Newman strain. In particular, three challenge models were used: the sepsis model, the skin model and the kidney abscess model. For the sepsis model, mice were immunized three times with 20 μg of CLSH-OMVs_Δ60_ and 14 days after the last immunization the animals received 3×10^8^ colony forming units (CFUs) of bacteria (i.p.). Animal health was followed every day over a period of seven days using a 1 to 4 pain scale (see *Material and Methods*). Animals that reached the pain value of 4 were sacrificed since they reached the “near mortality point” and would have died within 24 hours. On the other hand, those animals that maintained a score lower than 4, fully recovered within 7 days. **Figure 3A** reports the data of mice immunized with Alum alone, “empty” OMVs_Δ60_ and CLSH-OMVs_Δ60_. As shown in the figure, immunization with “empty” OMVs_Δ60_ conferred a certain level of protection, with 50% of the animals that did not reach pain value 4 and survived. By contrast, 100% protection was observed in the group of mice immunized with CLSH-OMVs_Δ60_ and none of the animals experienced a pain value higher than 2. The protective activity of the CLSH-OMVs_Δ60_ was also tested in the skin infection model and in the kidney abscess model. As shown in **Figures 3B, C**, CLSH-OMVs_Δ60_ vaccination was highly protective in both models. In particular, differently from Alum-immunized and “empty” OMVs_Δ60_-immunized animals, mice immunized with CLSH-OMVs_Δ60_ and subsequently s.c. challenged with 5×10^7^ CFUs developed only a mild and transient skin abscess with no dermonecrotic lesions. The differences between vaccinated and control animals was statistically significant (p < 0.001) as evaluated by means of repeated ANOVA measures using the R base function *aov()*. As far as the kidney abscess model is concerned, according to which 10^7^ CFUs were given intravenously and bacteria were counted in the kidneys four days after challenge, the CFUs counts in 13 out of 16 immunized mice were below the detection limit (10^2^ CFUs). As previously shown (9), immunization with “empty” OMVs_Δ60_ also resulted in a substantial level of protection (62.5%), in line with the protective role of an innate-type immune response induced by the OMVs.

### Four-Antigen Engineering of OMVs Can Be Successfully Applied to Other Antigen Combinations

**Figure 4:**
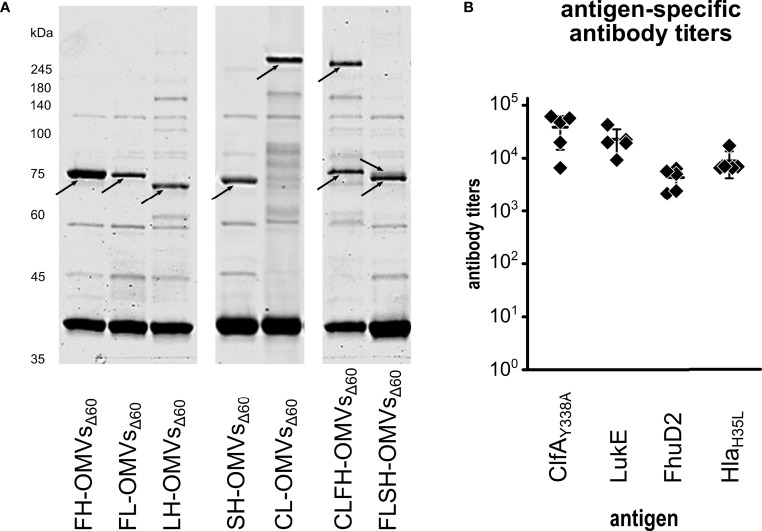
Expression of chimeras in OMVs_Δ60_. **(A)** *E. coli* BL21(DE3)Δ60 was transformed with plasmids expressing the following chimeras: FhuD2-Hla_H35L_, FhuD2-LukE, LukE-Hla_H35L_, SpA_KKAA_-Hla_H35L_ and ClfA_Y338A_-LukE. One recombinant clone from each transformation was inoculated in liquid culture and the corresponding OMVs, named FH-OMVs_Δ60_, FL-OMVs_Δ60_, LH-OMVs_Δ60_, SH-OMVs_Δ60_ and CL-OMVs_Δ60_, respectively, were purified from each culture supernatants. Purified OMVs were analyzed by SDS-PAGE (10 μg total OMV proteins) (left panel). The right panel reports the SDS-PAGE analysis of OMVs from *E. coli* BL21(DE3)Δ60 co-transformed with plasmids expressing either ClfA_Y338A_-LukE and FhuD2-Hla_H35L_ (CLFH-OMVs_Δ60_) or FhuD2-LukE and SpA_KKAA_-Hla_H35L_ (FLSH-OMVs_Δ60_) using pET21b(+) and pACYC, respectively (see *Materials and Methods* for details). **(B)** A group of five mice were immunized three times two weeks apart with CLFH-OMVs_Δ60_. After two weeks from the last immunization sera were collected and pooled and the ELISA titers were measured coating the plates with recombinant proteins.

The results described above demonstrate the feasibility of co-expressing the two chimeras SpA_KKAA_-Hla_H35L_ and ClfA_Y338A_-LukE in the same OMVs preparation. For a broad applicability of the technology it would be important to prove that OMVs can be successfully decorated with other chimera combinations. Therefore, we created three additional chimeras by combining LukE and Hla_H35L_ with FhuD2, another virulence factor involved in *S. aureus* pathogenesis (50), and by fusing together the two toxins LukE and Hla_H35L_. Three plasmids were generated pET(FhuD2-LukE), pACYC(FhuD2-Hla_H35L_) and pET(LukE-Hla_H35L_). The plasmids were used to transform *E. coli* BL21(DE3)Δ60 strain and one transformant colony of each transformation was used to produce OMVs. As shown in **Figure 4A**, the three chimeras efficiently accumulated in the vesicular compartment. Next, we co-transformed the strain with the plasmid couples pET(FhuD2-LukE)/pACYC(SpA_KKAA_-Hla_H35L_) and pET(ClfA_Y338A_-LukE)/pACYC(FhuD2-Hla_H35L_) and the successful compartmentalization of the two chimera couples in FLSH-OMVs_Δ60_ and CLFH-OMVs_Δ60_ was confirmed by SDS-PAGE (**Figure 4A**). Finally, we used CLFH-OMVs_Δ60_ to immunize mice and we analyzed the antibody titers against all antigens. As shown in **Figure 4B**, the engineered OMVs elicited antibodies specific for all four chimera antigens.

On the basis of these experiments we concluded that our OMV engineering strategy has a broad applicability and can be exploited for the preparation of multi-component vaccines against one or more pathogens.

## Discussion

One of the key issues in vaccinology is to find ways to make vaccine production as economic and easy as possible to guarantee that vaccines can reach out even the poorest people and to allow low income countries to establish their own production plans and distribute vaccines to their population.

In this respect, vaccines based on OMVs engineered with heterologous antigens appear to be attractive. Their built-in adjuvanticity and the ease with which they can be produced by using a single ultrafiltration step from bacterial culture supernatants make their production process inexpensive and simple. However, vaccines against recalcitrant pathogens usually require more than one antigen. This complicates the production process, even in the case of the OMV-based vaccines. Multi-component vaccines would require the purification of more than one engineered OMVs and their subsequent combination to make the final formulation (9).

In this work we describe a new strategy to co-express four antigens in the same OMVs, thus making the preparation of multi-component vaccines simple and inexpensive.

Using four important *S. aureus* virulence factors as a model, we first showed that OMVs can be engineered with two antigens by creating two-protein chimeras. Such chimeras can be efficiently expressed in the membrane of OMVs exploiting the lipoprotein transport machinery. From an immunological standpoint, protein fusions are attractive since each antigen of the fusion takes advantage of the additional T cell help provided by the T cell epitopes of the partner. This usually results in enhanced antigen-specific immunogenicity and antibody titers (51).

Next, we showed that if two chimeras are expressed from two plasmids with compatible origins of replication in the same OMV-producing strain, vesicles containing both chimeras can be obtained. In line with the evidence that the loading capacity of OMVs_Δ60_ is limited and cannot exceed a certain level (17), the expression of each co-expressed chimera was slightly reduced with respect to the same chimera expressed alone. However, since our OMV-producing strain has the unique property to release vesicles with high yield and improved loading capacity, being deprived of 60 endogenous proteins, the amount of each chimeric protein (approx. 5% of total OMV proteins) was sufficiently high to guarantee excellent immunogenicity. The immunization with 20 μg of CLSH-OMVs_Δ60_ elicited antibody titers against each antigen similar to what obtained with the combination of OMVs (5 μg each, 20 μg of total OMVs proteins) expressing the single antigens (9). Since we previously showed that 5 μg of OMVs engineered with single antigens are sufficient to reach the plateau of antigen-specific antibody titers, 20 μg of CLSH-OMVs_Δ60_ should represent an appropriate dose to elicit optimal immune responses.

To demonstrate the broad applicability of the approach, we created three additional chimeras and we showed that all of them could be successfully expressed in OMVs. Moreover, by using the two plasmid expression strategy, we confirmed that two chimeras could be efficiently co-expressed in the same OMVs and that such OMVs elicited immune responses against all four antigens. Therefore, we believe that our approach represents a step forward to make multi-component vaccines, including protective antigens from the same pathogen as well as from different pathogens.

A second important result of our work is the development of a new *S. aureus* vaccine candidate ready to move to development.

*S. aureus* is a Gram-positive organism, which colonizes the anterior nares and the skin of approximately one third of individuals at any time (16). Usually colonization is asymptomatic. However, in patients with immunological or barrier defects, *S. aureus* can become invasive and responsible for severe diseases (52). Moreover, highly pathogenic strains have recently emerged, many of which are resistant to most antibiotics and have the ability to cause diseases in otherwise healthy individuals (53). In the USA, approximately 3.4 million community-acquired diseases and 340.000 hospital acquired-diseases occur annually, leading to more than 30.000 deaths (54).

When *S. aureus* becomes invasive, it expresses more than 35 immune evasion molecules and various virulence factors (12). Moreover, once phagocytosed, *S. aureus* has the ability to avoid killing, thus using phagocytes as “Trojan Horses” to disseminate itself inside the host (15, 16). This peculiarity makes the development of an effective vaccine particularly challenging. Three Phase III *S. aureus* vaccine trials have been reported (55–57). However, the efficacy data of these vaccines, all formulated without adjuvants, were largely disappointing. The vaccine failures can be attributed to an inappropriate antigen selection in terms of both quality and quantity. In this respect, the redundancy of virulent factors expressed by the different *S. aureus* isolates represents a serious challenge in establishing which antigens are necessary and sufficient to elicit a broadly protective immunity. Moreover, to counteract the ability of *S. aureus* to survive inside host cells, a vaccine should elicit a Th1/Th17-skewed adaptive immune response and a strong innate immunity, the latter necessary to enhance the killing capacity of phagocytic cells. To achieve this, a vaccine should combine protective antigens with a proper adjuvant and, to keep innate immunity constantly alerted, the vaccine should be administered repeatedly, particularly when the risk of infection increases, for instance during hospitalization.

We believe that CLSH-OMVs_Δ60_ have many properties that make them attractive as vaccine candidate.

The vaccine elicits both opsonophagocytic antibodies and functional antibodies against four important virulence factors. Opsonophagocytosis is considered to be an important prerequisite of *S. aureus* vaccines (55–57), and the sera from mice immunized with CLSH-OMVs_Δ60_ have the ability of killing *S. aureus in vitro* in the presence of phagocytic cells and complement. Such killing activity is likely to be mediated by the recognition of SpA and ClfA, which are both surface-exposed proteins. Moreover, anti-CLSH-OMVs_Δ60_ antibodies inhibited the cytotoxic activity of Hla and LukED, two potent toxins that *S. aureus* releases to damage tissues and to kill different subsets of immune cells. Finally, the sera from immunized animals neutralized the fibrinogen-mediated adhesion of *S. aureus* to tissues and, although not directly tested, are expected to inhibit the SpA-specific binding to immunoglobulins as previously described (23, 26, 27).

The single OMV vaccine here described appears to provide a protection which is at least as good as the protection we observed with our five-OMVs formulation, which included five vesicles each engineered with one of the antigens Hla_H35L_, LukE, SpA_KKAA_, FhuD2 and CsaA1 (9). Interestingly, our new candidate shares three antigens with the previously published vaccine (9), but differs for the replacement of Csa1A and FhuD2 with ClfA_Y338A_. This data would corroborate the notion that ClfA is an important virulence factor whose inhibition is particularly relevant in the early stage of infection (32).

A second important property of our CLSH-OMVs_Δ60_ vaccine is its capacity to elicit a Th1/Th17-skewed adaptive immunity and a strong innate immunity. According to our opinion, this is an important feature since, to be efficiently neutralized, *S. aureus*, which has the unique capacity to survive within phagocytic cells, should encounter a full-blown adaptive and innate immunity at the time of the host invasion. This would imply that CLSH-OMVs_Δ60_ should be administered whenever the risk of infection increases, as it is for instance the case of hospitalized patients awaiting surgery.

The role of vaccine-induced innate immunity is gaining increasing attention in the light of the evidence that certain infections and vaccinations can induce a broad protection against other pathogens through innate immune mechanisms (58, 59). Strikingly, innate immunity appears to have memory characteristics (referred to as “trained innate immunity”), a feature previously thought to be an exquisite property of adaptive immunity [for an excellent review, see (60)]. The role and the duration of OMV-induced innate immunity in humans is certainly a topic of great interest that should be addressed in future clinical trials.

## Data Availability Statement

The original contributions presented in the study are included in the article/**Supplementary Material**. Further inquiries can be directed to the corresponding author.

## Ethics Statement

The animal study was reviewed and approved by the Animal Ethical Committees of the University of Trento, the Animal Ethics Committees of Toscana Life Sciences and the Italian Ministry of Health.

## Author Contributions

GG conceived and designed the study, analyzed data and wrote the manuscript. EK, IR, CA, and CI: OMV engineering. EK, IR, CA, LF, IZ, and EC: OMV production and purification. EK, AGr, MT, LFa, and AG: OMV immunogenicity. MT and LFa: mouse models. AG, AGr and FB: OPK assay. AG and AGr: neutralization assays for ClfA, LukE and Hla. EK, AG, and MT: ELISA. EK and AG: cloning and purification of recombinant proteins. EK and AG edited manuscript and Figures. All authors contributed to the article and approved the submitted version.

## Funding

The work has been financially supported by the Advanced European Research Council grant OMVAC 340915 and the Advanced European Research Council grant Vaccibiome 834634 (both assigned to GG).

## Conflict of Interest

Author FB was employed by company GlaxoSmithKline Vaccines. GG, EK, IZ, CI, and LFa are coinventors of a patent on OMVs. AGr and GG are involved in a biotech company interested in exploiting the OMV platform.

The remaining authors declare that the research was conducted in the absence of any commercial or financial relationships that could be construed as a potential conflict of interest.

## Publisher’s Note

All claims expressed in this article are solely those of the authors and do not necessarily represent those of their affiliated organizations, or those of the publisher, the editors and the reviewers. Any product that may be evaluated in this article, or claim that may be made by its manufacturer, is not guaranteed or endorsed by the publisher.

## Supplementary Material

Supplementary Figure 1
*Analysis of antigen-specific antibody titer curves.* Sera from mice immunized i.p. with CLSH-OMVs_Δ60_ (squares) or “empty” OMVs_Δ60_ (triangles) both with Alum were tested for antibody titers by ELISA using plates coated with recombinant proteins (300 ng/well). Each data point represents the media (n = 8) of absorbance at 405 nm at the respective serum dilution. Mean ± s.e.m. is shown.

Supplementary Figure 2
*Antigen-specific antibody titers of IgG isotypes.* Sera from mice immunized i.p. with CLSH-OMVs_Δ60_ + Alum were tested for antibody titers of IgG isotypes (IgG2a and IgG1) by ELISA using plates coated with recombinant ClfA_Y338A_ and SpA_KKAA_ (300 ng/well). Each data point represents the antibody titer from a single mouse. Geometric mean ± 95% CI is shown.
