# Supplementary figures and images for: Multi-Antigen Outer Membrane Vesicle Engineering to Develop Polyvalent Vaccines: The Staphylococcus aureus Case
Source: Front Immunol. 2021 Nov 8;12:752168. doi: 10.3389/fimmu.2021.752168 (PMC8606680; doi:10.3389/fimmu.2021.752168)

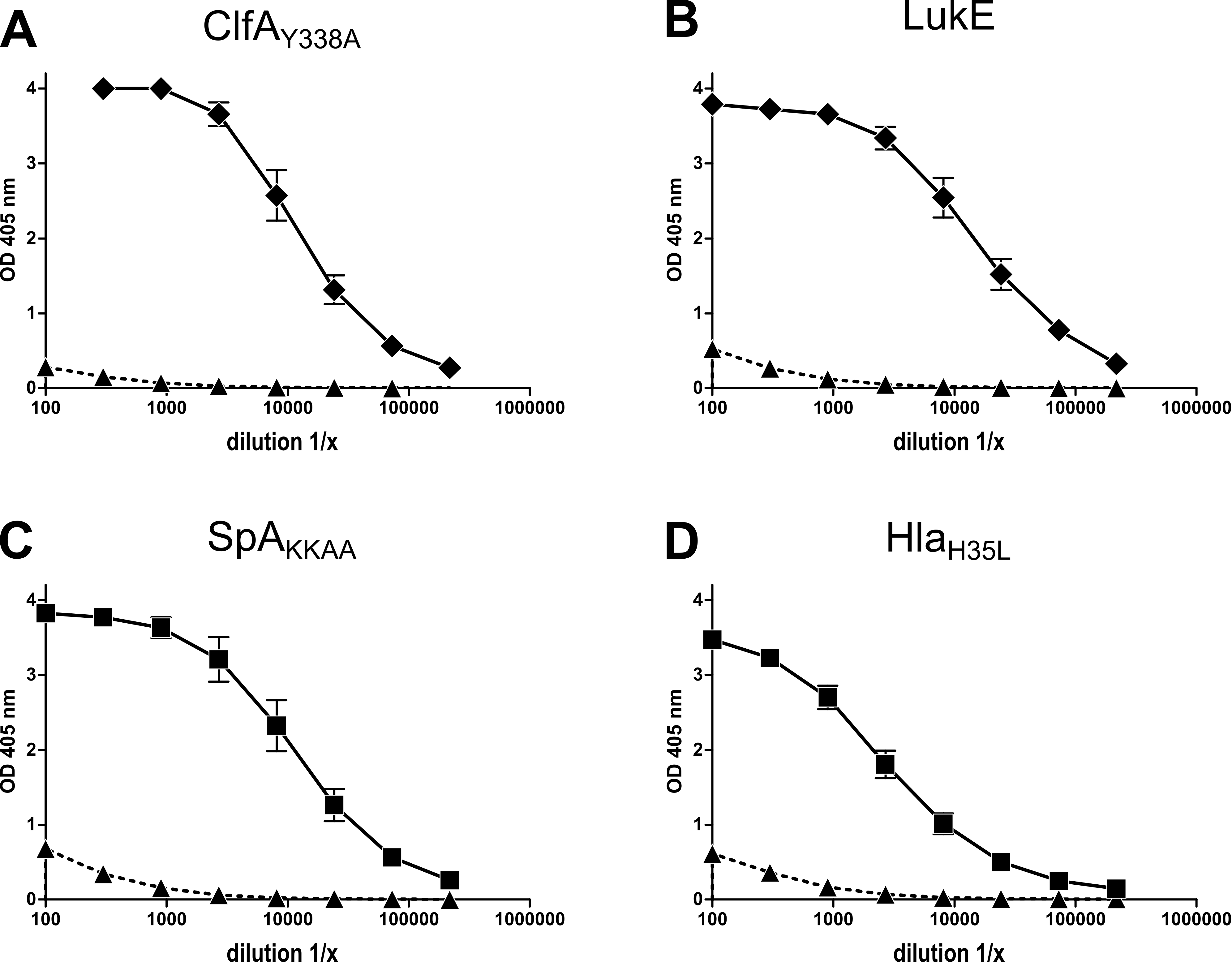

Supplement: Supplementary Figure 1 — Analysis of antigen-specific antibody titer curves. Sera from mice immunized i.p. with CLSH-OMVsΔ60 (squares) or “empty” OMVsΔ60 (triangles) both with Alum were tested for antibody titers by ELISA using plates coated with recombinant proteins (300 ng/well). Each data point represents the media (n = 8) of absorbance at 405 nm at the respective serum dilution. Mean ± s.e.m. is shown. [file Image_1.jpeg]

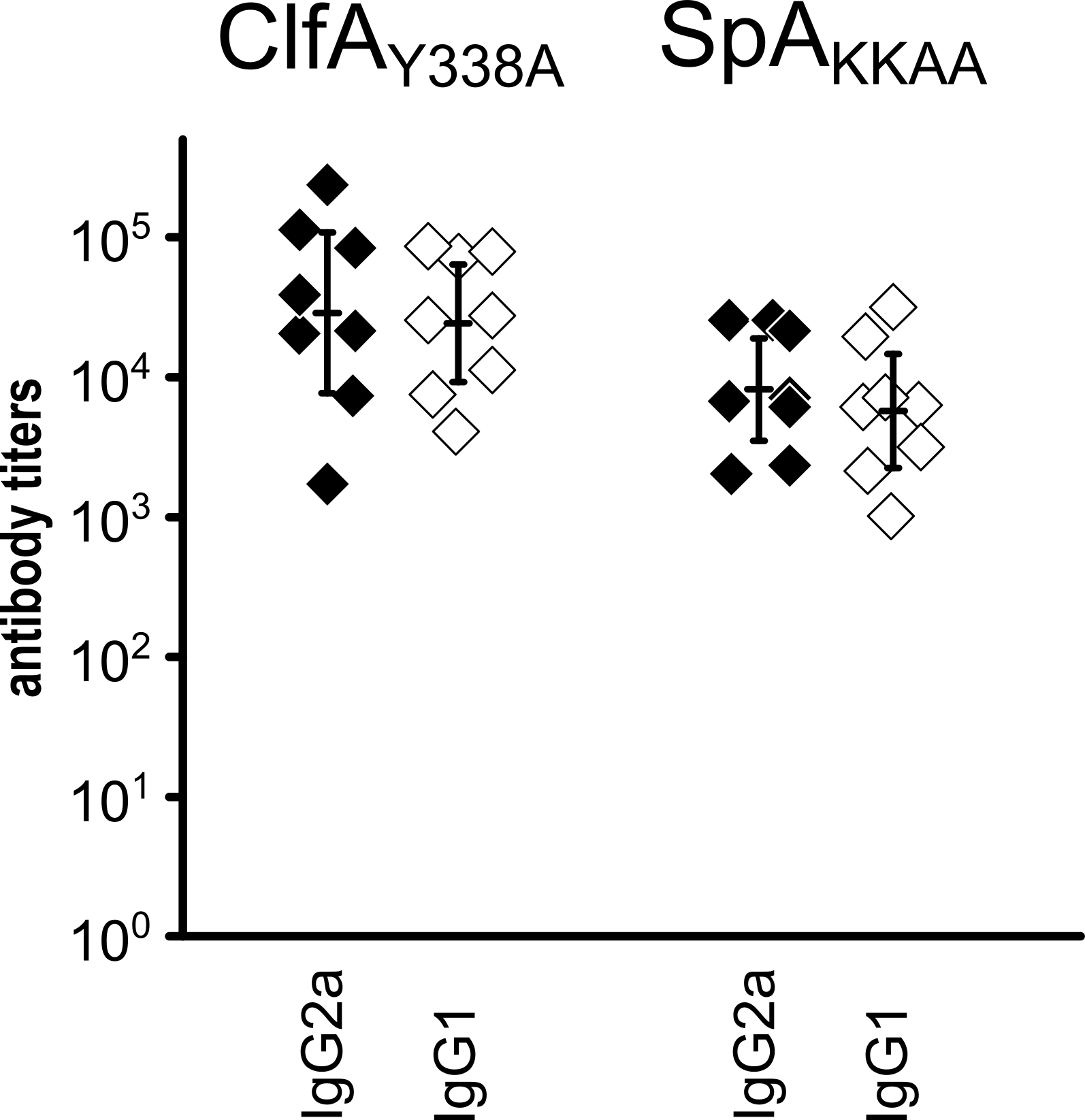

Supplement: Supplementary Figure 2 — Antigen-specific antibody titers of IgG isotypes. Sera from mice immunized i.p. with CLSH-OMVsΔ60 + Alum were tested for antibody titers of IgG isotypes (IgG2a and IgG1) by ELISA using plates coated with recombinant ClfAY338A and SpAKKAA (300 ng/well). Each data point represents the antibody titer from a single mouse. Geometric mean ± 95% CI is shown. [file Image_2.jpeg]
